# Genomic regions linked to alcohol consumption in the Framingham Heart Study

**DOI:** 10.1186/1471-2156-4-S1-S101

**Published:** 2003-12-31

**Authors:** Andrew W Bergen, Xiaohong Rose Yang, Yan Bai, Michael B Beerman, Alisa M Goldstein, Lynn R Goldin

**Affiliations:** 1 Core Genotyping Facility, Advanced Technology Center, National Cancer Institute, NIH, DHHS, Gaithersburg, Maryland, USA; 2 Genetic Epidemiology Branch, Division of Cancer Epidemiology and Genetics, National Cancer Institute, NIH, DHHS, Bethesda, Maryland, USA; 3 Core Genotyping Facility, SAIC-Frederick, Inc., Frederick, Maryland, USA

## Abstract

**Background:**

Pedigree, demographic, square-root transformed maximum alcohol (SRMAXAPD) and maximum cigarette (MAXCPD) consumption, and genome-wide scan data from the Framingham Heart Study (FHS) were used to investigate genetic factors that may affect alcohol and cigarette consumption in this population-based sample.

**Results:**

A significant sister:sister correlation greater than spouse correlation was observed for MAXCPD only. Single-point sib-pair regression analysis provided nominal evidence for linkage of loci to both SRMAXAPD and MAXCPD consumption traits, with more significant evidence of linkage to SRMAXAPD than to MAXCPD. One genomic region, chr9q21.11, exhibits significant multi-point sib-pair regression to SRMAXAPD.

**Conclusion:**

SRMAXAPD exhibits greater evidence for genetic linkage than does MAXCPD in the FHS sample. Four regions of the genome exhibiting nominal evidence for linkage to SRMAXAPD in the FHS sample correspond to regions of the genome previously identified as linked to alcoholism or related traits in the family data set ascertained on individuals affected with alcohol dependence known as COGA.

## Background

Data from the ongoing NHLBI-supported Framingham Heart Study (FHS) on cardiovascular disease (CVD) was made available to Genetic Analysis Workshop 13 (GAW13). Two behaviors of general medical and psychiatric interest collected from this community-based sample were included in the data, i.e., alcohol consumption and cigarette consumption. Increased cigarette consumption in the FHS sample is associated with the development of CVD [1,2], but increased alcohol consumption in the FHS is not, except in those aged 60–69 [3,4], although meta-analyses of cohort and case-control samples, including the FHS [3], identify a protective effect of moderate (1–2 drinks/day) alcohol consumption [5]. The consumption of tobacco and alcohol confer significant risk for a variety of medical disorders other than CVD, e.g., oral and pharyngeal cancer [6] and for a common psychiatric comorbidity [7].

The consumption of these two substances varies significantly based on both sex and age and there has been a long-term decline in the consumption of cigarettes in the US in the latter half of the 20^th ^century, due to health concerns and restrictions placed on this behavior [8]. The consumption of both substances is significantly correlated in the American population and the prevalence of consumption of alcohol and tobacco is increased by a factor of two in consumers of either substance [9]. The genetic influence on alcohol and tobacco dependence is significantly correlated in men [10]. Measures of consumption in multiple exams of the FHS provide an opportunity to study the genetic correlation of alcohol and tobacco consumption traits and search for susceptibility loci for these traits in a community-based sample.

## Results

### Descriptive analysis of MAXAPD and MAXCPD

There were a total of 4692 individuals in the GAW13 FHS sample, 2849 with a maximum alcohol consumption (MAXAPD) measure and 2881 with a maximum cigarette consumption (MAXCPD) measure. Descriptive statistics of the MAXAPD and MAXCPD traits and a square root transformation of MAXAPD, SRMAXAPD, are reported in Table 1. The maximum alcohol consumption traits are highly non-normal and the distribution remained highly non-normal whether or not individuals with APD = 0 (*N *= 346) were included. Individual outlier trait values > +4 standard deviations were changed to missing for analysis (without outliers); the individuals whose values were converted were predominantly male for all three traits but were mostly (≥80%) from Cohort 1 for APD traits (*N *= 28 for MAXAPD, *N *= 7 for SRMAXAPD) and from Cohort 2 for MAXCPD (*N *= 10). Removal of outliers brings SRMAXAPD and MAXCPD traits much closer to normality (Table 2). Because MAXAPD remains highly non-normal even after positive outlier removal (Table 2), correlation and linkage analysis results with MAXAPD are not reported.

### Correlations of alcohol and cigarette consumption traits

Familial correlations of relative and parental pairs (sex-specific and non-sex-specific) without extreme positive outliers for MAXCPD and SRMAXAPD are reported in Table 3. For MAXCPD, only the correlation between sisters is greater than that between parents, with or without outliers. There are no familial correlations greater than the spousal correlation for SRMAXAPD, with or without outliers.

### Sib-pair linkage analyses

**Figure 1:**
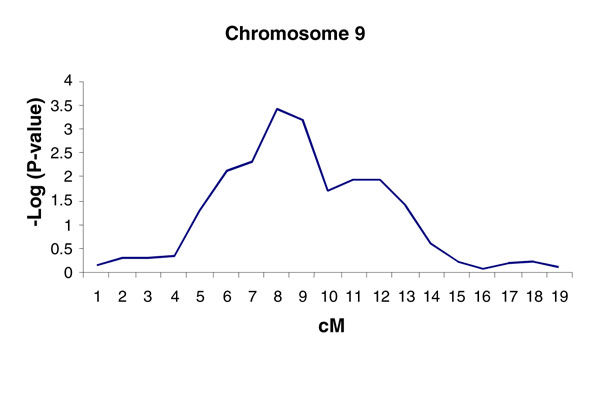
Multi-point linkage analysis of SRMAXAPD (outliers removed).

In the single-point analysis, SRMAXAPD shows nominal evidence for linkage at 17 markers on 10 chromosomes with a *p*-value < 0.01 (Table 4). Chromosomes 7 and 9 have 3 markers each significant at a *p*-value < 0.01. In addition, for this analysis treating sex as a covariate significantly predicted sib-pair trait covariance at *p *< 0.01, while the regression analysis of age at which the maximum amount of alcohol was consumed and cohort did not provide nominal evidence for linkage at the *p *< 0.05 level. Multi-point linkage analysis with SRMAXAPD identified chromosome 9 as the chromosome with the most significant multi-point linkage result in the 10 chromosomes evaluated. Thirteen consecutive markers on chromosome 9 showed evidence of linkage at *p *< 0.05, with five of them showing significance at *p *< 0.01. The most significant *p*-value observed on chromosome 9 was 3.77 × 10^-4 ^at marker c9g8 (Figure 1).

The single-point analysis for the trait for cigarette consumption, MAXCPD, found nominal evidence for linkage at nine markers on 7 chromosomes at *p *< 0.05 (Table 5). The effects of the covariates cohort, sex, and age at MAXCPD on sib-pair trait covariance were inconsistent. There was only one chromosome with more than one marker exhibiting nominal evidence for linkage. Multi-point results for MAXCPD were unremarkable.

## Discussion

In the genome-wide search for linkage evidence to maximum alcohol consumption, we found a number (*N *= 17) of marker loci that were nominally linked (*p *< 0.01) with the maximum alcohol consumption traits, SRMAXAPD. A broad region on chromosome 9 exhibited the most significant evidence for linkage, with the maximum linkage evidence at ~66 cM (Table 4 and Figure 1). For the cigarette consumption trait, MAXCPD, we observed fewer loci (*N *= 9) with evidence for nominally significant (*p *< 0.05) linkage and no loci at *p *< 0.021. The low number of loci exhibiting nominal evidence for linkage to MAXCPD suggests that the MAXCPD trait, as investigated in this linkage analysis, lacks power to detect the influence of genetic susceptibility factors on maximum cigarette consumption.

Empirical *p*-values for the significance of linkage analysis results were not substantially different from asymptotic *p*-values for either trait, suggesting that assumptions of the SIBPAL regression model apply to the phenotypic and genotypic data analyzed in this study. Only the regions of maximum linkage to SRMAXAPD on chromosome 9 and the single-point linkage analysis result to SRMAXAPD on chromosome 17 at 89 cM provided statistical evidence for linkage at *p *< 0.0007, a level considered "significant" by Lander and Kruglyak [11]. Multiple testing corrections in investigations of alcohol and cigarette consumption phenotypes performed independently, as in this study, would need to consider the significant correlation between the two behaviors [9].

Linkage studies of alcohol- and cigarette-related traits have identified regions of the genome with more than nominal evidence for linkage [12]. Regions of the genome that have been identified as nominally linked to phenotypes related to alcohol consumption include chromosome 1 (~170 cM) and chromosome 7 (~80–100 cM) for alcohol dependence [13,14], chromosome 1 (~200–250 cM) and chromosome 15 (~70 cM) with a factor composed of later age of onset of drinking and increased harm avoidance [15], chromosome 4 (~120 cM) for alcohol consumption [16], chromosome 1 (~100–150 cM) for alcoholism or depression [17], chromosome 1 (~100–150 cM) and chromosome 21 (~80 cM) for alcohol sensitivity [18], all in the COGA sample [13], and chromosome 4 (~70 cM) and chromosome 11 (~0 cM) in the NIDDK/NIAAA American Indian sample [19]. Regions identified in this analysis of maximum alcohol consumption in the Framingham sample that correspond to the regions identified in the literature include chromosomes 1 [15], 4 [18], 7 [16], and 15 [15]. Regions of the genome that have been identified as nominally linked to phenotypes related to cigarette consumption in other samples include chromosome 1 (~0 cM), chromosome 2 (~90 cM), chromosome 14 (~95 cM) for ever-smoking in the COGA sample [20], and chromosome 2 (~145 cM) and chromosome 10 (~120 cM) in the Christchurch sample [21]. There were no regions of the genome in the Framingham sample with nominal evidence of linkage to cigarette consumption that overlapped regions identified in the COGA and Christchurch samples exhibiting linkage to cigarette related phenotypes. However, in the FHS sample, chromosome 15 contains markers that exhibit nominal evidence for linkage to SRMAXAPD (Table 4) and MAXCPD (Table 5) at 60 cM and 72 cM, close to a region of chromosome 15 in the COGA sample exhibiting suggestive linkage to a factor composed of later age of onset of drinking and increased harm avoidance [15].

## Conclusions

We observed several marker loci with nominal evidence for linkage to the square-root transformed maximum alcohol consumption traits, SRMAXAPD, in the Framingham Heart Study sample. Some of the regions of the genome have been previously linked to alcoholism or related traits in Caucasian samples based on different ascertainment criteria.

## Methods

### Selection of consumption data

The traits of interest were defined as the maximum reported number of grams of alcohol per day (MAXAPD), and the maximum reported number of cigarettes smoked per day (MAXCPD). Exams 1, 2, 4, and 7 from FHS Cohort 1 and exams 1, 2, 3, and 4 from FHS Cohort 2 were chosen to assess MAXAPD and MAXCPD to utilize multiple exams at the earliest age possible to obtain measurements. Covariates of interest included cohort, age of maximum consumption measure, and sex.

### Analysis of phenotypic, pedigree, and genotypic data

GAW13 FHS data were imported into a Microsoft Access database and exported in appropriate files for descriptive statistical analysis in SPSS Advanced Statistics or Microsoft Excel and familial correlation and linkage analysis in S.A.G.E. [22]. Familial correlations and the asymptotic standard errors were estimated using FCOR from S.A.G.E. 4.2. We used GENIBD from S.A.G.E. 4.2 to generate identity-by-descent (IBD) sharing distributions of the GAW13 data. Before running the GENIBD analysis, we used MEGA2 [23] to convert the GAW13 data to the column-delimited S.A.G.E. format.

SIBPAL from S.A.G.E. 4.2 was used to model the sib-pair covariance of traits reported as a function of marker allele identity-by-descent (IBD) sharing. Our analyses used estimated IBD information from the GENIBD procedure described above to perform single-point linkage analysis in which the mean corrected cross product of the trait was regressed onto the IBD information one trait at a time. The single-point linkage analysis was carried out separately for traits MAXAPD, SRMAXAPD, and MAXCPD, treated as continuous variables. The covariates sex, age of trait report, and cohort were included in the regression models. SRMAXAPD single-point linkage analysis was only performed without outliers. Empirical *p*-values were obtained for single-point linkage analysis of MAXCPD and SRMAXAPD to evaluate possible deviation from asymptotic *p-*values. Multi-point linkage analysis was performed using IBD distributions at multiple markers for MAXCPD and SRMAXAPD on those chromosomes showing nominal evidence (*p *< 0.05 and *p *< 0.01, respectively) for linkage at two or more consecutive loci.

## Authors' contributions

AB nominated the traits, performed the descriptive and FCOR analyses, and wrote the manuscript, XY performed the linkage analysis of the alcohol consumption traits, YB performed the linkage analysis of the cigarette consumption trait, MB performed the GAW13 FHS databasing, selection, reduction, transformation, and export, and AG and LG provided analysis direction and recommendations at each phase of the analysis.

## Figures and Tables

**Table 1:** Non-normality of MAXAPD, SRMAXAPD, and MAXCPD, with outliers.

Trait	*N*	Min	Max	Mean	Median	SD	Kurtosis	SE	Skewness	SE
MAXAPD	2849	0	624.00	23.34	11.00	35.80	57.88	0.09	5.39	0.05
SRMAXAPD	2849	0	24.98	3.86	3.32	2.91	2.82	0.09	1.14	0.05
MAXCPD	2881	0	95.00	13.93	10.00	15.61	0.96	0.09	1.06	0.05

**Table 2:** Non-normality of MAXAPD, SRMAXAPD, and MAXCPD, without outliers.

Trait	*N*	Min	Max	Mean	Median	SD	Kurtosis	SE	Skewness	SE
MAXAPD	2821	0	166.00	21.15	11.00	26.25	5.64	0.09	2.14	0.05
SRMAXAPD	2842	0	15.43	3.82	3.32	2.80	0.77	0.09	0.84	0.05
MAXCPD	2871	0	70.00	13.69	10.00	15.08	-0.03	0.09	0.88	0.05

**Table 3:** Significant familial correlations of SRMAXAPD and MAXCPD without outliers.

Correlation	MAXCPD	± SE	SRMAXAPD	± SE
father:son	0.058	0.041	0.133	0.041
mother:son	0.018	0.042	0.097	0.039
father:daughter	0.153	0.041	0.105	0.041
mother:daughter	0.141	0.041	0.163	0.039
brother:brother	0.179	0.045	0.150	0.046
sister:brother	0.130	0.034	0.164	0.032
sister:sister	0.268	0.046	0.186	0.045
parent:offspring	0.072	0.023	0.102	0.022
sibling:sibling	0.171	0.026	0.131	0.025
mother:father	0.199	0.044	0.310	0.042

**Table 4:** Markers exhibiting evidence (*p*-value < 0.01) for linkage to SRMAXAPD.^A^

Loci	Marker	Pairs	Regression Estimate	SE	*p-*Value
c1g27	D1S549	1455	1.55	0.54	0.0023
c4g3	D4S2633	1373	1.54	0.55	0.0025
c4g7	D4S3248	1432	1.47	0.57	0.0051
c6g10	D6S1053	1478	1.37	0.54	0.0054
c6g14	D6S1021	1428	1.42	0.57	0.0061
c7g10	D7S2204	1448	1.43	0.51	0.0027
c7g16	D7S1804	1426	1.34	0.52	0.0049
c7g22	D7S559	1382	1.63	0.52	0.0010
c9g8	D9S301	1410	1.75	0.53	0.0005
c9g9	D9S1122	1458	1.50	0.59	0.0056
c9g13	D9S910	1499	1.57	0.60	0.0044
c12g2	Unknown	1340	1.30	0.52	0.0061
c12g11	D12S1052	1458	1.35	0.56	0.0084
c15g7	D15S1507	1406	1.44	0.58	0.0064
c16g13	D16S539	1465	1.44	0.55	0.0046
c17g12	D17S1290	1485	1.63	0.50	0.0006
c22g4	D22S689	1470	1.39	0.55	0.0058

^A^Linkage analysis via regression of the mean corrected cross product of SRMAXAPD to allele IBD information with covariates sex, age at SRMAXAPD, and cohort.

**Table 5:** Markers exhibiting evidence (*p*-value < 0.05) for linkage to MAXCPD.^A^

Loci	Marker	Pairs	Regression Estimate	SE	*p*-Value
c5g14	D5S1726	306	79.40	38.79	0.021
c6g1	Unknown	1425	43.24	23.82	0.035
c6g20	242zg51	1444	40.36	21.61	0.031
c8g3	D8S1130	1420	45.96	22.89	0.022
c9g18	308vb11	313	94.17	55.44	0.045
c15g9	D15S818	1103	50.77	28.19	0.036
c16g4	049xd21	1476	37.49	21.63	0.042
c20g9	321xd11	300	84.67	43.70	0.027
c20g10	D20S480	1412	41.11	23.86	0.043

^A^Linkage analysis via regression of the mean corrected cross product of MAXCPD to allele IBD information with covariates sex, age at MAXCPD, and cohort.
